# Evaluation of the maternal mortality surveillance system in Mutare district, Zimbabwe, 2014-2015: a cross sectional study

**DOI:** 10.11604/pamj.2017.27.204.7210

**Published:** 2017-07-18

**Authors:** Faith Mutsigiri-Murewanhema, Patron Trish Mafaune, Tsitsi Juru, Notion Tafara Gombe, Donewell Bangure, More Mungati, Mufuta Tshimanga

**Affiliations:** 1 Department of Community Medicine, University of Zimbabwe, Zimbabwe; 2 Ministry of Health and Child Care, Zimbabwe

**Keywords:** Maternal mortality, surveillance, Mutare district, Zimbabwe

## Abstract

**Introduction:**

In Zimbabwe the integrated disease surveillance and response guidelines include maternal mortality as a notifiable event reported through the Maternal Mortality Surveillance System (MMSS). A preliminary review of the MMSS data for Mutare district for the period January to June 2014 revealed that there were some discrepancies in cases notified and those captured on the T5 monthly return form. There were also delays in reporting of some maternal deaths. Poor reporting indicated shortcomings in the MMSS in Mutare district and we therefore sought to assess the performance of the maternal mortality surveillance system in Mutare district.

**Methods:**

A descriptive cross sectional study was conducted using Centers for Disease Control and Prevention updated guidelines for evaluating public health surveillance systems. A total of 64 health workers were enrolled into the study from 19 selected health facilities in Mutare district and 32 maternal death notification forms submitted in 2014 to the provincial office were reviewed to assess the quality of information on the forms. Interviewer administered questionnaires were used to collect information from enrolled health workers, the system's attributes namely usefulness, acceptability, simplicity, stability, data quality, timeliness and completeness were assessed and a checklist was used to assess availability of resources for the implementation of the maternal mortality surveillance. We also determined the cost of reporting each maternal death in Mutare district.

**Results:**

Half of the study participants gave the correct definition of a maternal death. All health workers participated and were willing to continue participating in the maternal mortality surveillance. Majority of health workers, 79.7% used data generated from the surveillance system and 59.5% found it easy to implement the system. A total of 32 death notification forms were reviewed and of these, 31 forms were forwarded to the national office and all did not reach the national office on time. Average completeness of notification forms was 76.0% and 53.1% of the forms had all the necessary accompanying documents. Reporting each maternal death was estimated to cost $28.65 in Mutare district.

**Conclusion:**

The strongest components of the maternal mortality surveillance system in Mutare district were usefulness and acceptability. Timeliness and completeness were the weaker components of the system. The system was found to be simple; however, resources were not adequately available in all health facilities.

## Introduction

Of all the Millennium Development Goals (MDGs), improving maternal health is the least likely to be achieved [1–3]. Despite knowledge on determinants and causes, and effective clinical and public health strategies [4]. The goal of reducing the Maternal Mortality Rate between 1990 and 2015 by three quarters is unlikely to be met. According to the World Health Organization, globally, approximately 800 women die from preventable causes related to pregnancy and childbirth each day. An estimated 287 000 women worldwide died from pregnancy and its complications in 2010 [5]. Developing countries account for 99% of the global maternal deaths, the majority (56%) of which are in Sub-Saharan Africa. The burden is more pronounced in poor, rural areas with young adolescents facing higher risk of complications and death as a result of pregnancy than older women [6]. In 2012 the United Nations Commission on the status of women passed on a resolution calling for the elimination of preventable maternal mortality. To accomplish this it is essential to have a system that measures and tracks all maternal deaths in real time, helps us understand the underlying factors contributing to the deaths and stimulates and guides actions to prevent future deaths. Maternal Deaths Surveillance and Response (MDSR) is a model of such a system. It stresses the need to respond to each maternal death with actions to prevent similar deaths in the future and to collect data on all maternal deaths using clearly defined data sources and processes for identification and notification [5]. Maternal deaths indicate weakness in the health care system [7]. Timely reporting of maternal deaths is critical if the MDSR system is to be successful-memories of such events and the surrounding circumstances becomes less clear as time passes [5].

In Zimbabwe Integrated Disease Surveillance and Response technical guidelines include maternal mortality as a notifiable condition reported through the Maternal Mortality Surveillance System (MMSS). Obligatory notification makes maternal mortality a national priority, underlining the fact that every maternal death matters. Many countries have already implemented a policy of adding maternal death to the list of notifiable diseases [8]. Generally classifying an event as notifiable means it must be reported to the authorities within 24 hours and followed up by a more thorough report of medical causes and contributing factors. This approach is used for many infectious diseases. Deaths occurring in health care facilities should be notified within 24 hours and deaths in the communities should ideally be notified within 48 hours. Immediate notification should be followed by a more thorough report [5]. Notification should be systematic, including absence of cases (zero reporting). Zero reporting means there is an active process of notifying suspected maternal deaths, whether or not any death occurred. If no maternal deaths occurred, a “zero” is captured in the data collection system rather than nothing at all. If an entry is left blank, it is not clear that attention was paid to the entry. Zero reporting is used as an indicator that active monitoring for maternal deaths is taking place. It helps remind people to look for these events [5]. In order to identify the levels and determinants of maternal mortality and emphasize the message that no maternal death is acceptable; all maternal deaths must be reviewed. Reviews should be done at the health facility or district level by multidisciplinary committees made up of health professionals and management staff. This is essential for changing practices, fostering ownership, and ensuring the best possible data quality [5].

**Maternal mortality surveillance system in Zimbabwe**: When a maternal death occurs, the next level should be informed within 24 hours by phone. Maternal death notification forms are completed in triplicate within seven days of the maternal death and one copy is retained at the reporting institution. A preliminary audit is recommended within seven days of the maternal death to determine the causes of the death. Audit results together with two maternal death notification forms, patient's notes and incidence report should reach the Provincial Medical Director's (PMD) office within 14 days of the maternal death. The provincial medical director's office will complete relevant sections of both forms and submit one copy to the reproductive health unit within 30 days of the death. Feedback on the death comes from the head office through the PMD's office then to the reporting institution through the district office. In order to end preventable maternal deaths, accurate information on how many women died, where they died and how they died is essential, but is currently inadequate. The maternal death notification system is complementary to the national Health Management Information System and is aimed at improving tracking, monitoring and reporting of maternal mortality. The MMSS should have 100% timeliness and completeness. However, the surveillance system in Mutare district failed to meet its target of 100% for both timeliness and completeness. A preliminary review of the MMSS data for the period January to June 2014 revealed that there were some discrepancies in cases notified & those captured on the T5. There were also delays in reporting of some maternal deaths. Poor reporting indicated shortcomings in the MMSS in Mutare District. The goal of the MMSS is to reduce maternal deaths by providing timely, quality information to identify causes of maternal deaths and inform remedial actions needed to prevent future maternal deaths. However, if data is of poor quality, more women will continue to die the same way from avoidable factors. Therefore, we sought to assess the performance of the MMSS in Mutare district.

## Methods

A descriptive cross sectional study using centers for disease control and prevention updated guidelines for evaluating public health surveillance systems was conducted. A total of 19 health facilities were included in the study and 64 health workers were interviewed. All major hospitals in Mutare district were purposively selected into the study. Simple random sampling was employed in the selection of the other health centers that participated in the evaluation. All heath workers (nurses and doctors) who were on duty on the day of the evaluation at the selected health institutions were eligible to participate in the study. Proportionate sampling was used to determine the number of health workers to be interviewed at each selected facility. Simple random sampling was employed to select health workers that were interviewed at each health facility. An interviewer administered questionnaire was used to collect information on health workers' knowledge of the MMSS, usefulness of the surveillance system, assessment of the system's attributes and to establish the cost of reporting each maternal death. A checklist was used to assess availability of resources for MMSS at health facilities. Permission to carry out the study was sought and granted by the health studies office, the provincial medical director (Manicaland province) and the district medical officer (Mutare district). Written informed consent was sought from all study participants and voluntary participation was ensured. No names were used on the questionnaires and the information was only available to those who were involved in the study. Data were analyzed using Epi info version 3.5.3 to calculate frequencies, means and proportions.

## Results

Sixty-four health workers were interviewed during the study. Fifty-one (79.7%) of these were female. The median age of health workers was 32 years (Q_1_=32; Q_3_=44.5). The majority of health workers (65.6%) were Registered Nurses and the median duration in service was 8 years (Q_1_=5; Q_3_=14.5). Half of the health workers gave the correct definition of a maternal death. Most health workers, 73.4% mentioned that the objective of the MMSS was to provide information that guides to eliminate preventable maternal deaths. Knowledge of the data collection tools and reporting timeframes was poor among health workers. Table 1 shows knowledge of the maternal mortality surveillance system among health workers in Mutare district, 2015. All health workers participated and were willing to continue participating in the MMSS. Table 2 summarizes the roles played by health workers in the MMSS, how they learnt to implement the MMSS and reasons for failing to compile surveillance data at times. Fifty-one (79.7%) health workers mentioned that they use data generated from the MMSS. Uses of this information were; making informed decisions on maternal health as stated by 35.3% of health workers, 25.5% used it for monitoring performance of public health interventions and 23.5% for capturing maternal mortality statistics. Table 3 shows usefulness of the MMSS. The majority, 82.8% held meetings to review each maternal death. The majority, 59.4% of health workers reported that it was easy to implement the maternal mortality surveillance system. However, 40.6% said it was difficult and the challenge mentioned by most, 13(50%), was the issue of religious objectors of the Apostolic sect in Mutare district who do not use health facilities for maternal health services and if a death occurs in their communities they do not report it. Too much time spend filling in maternal deaths notification forms was reported by 26.9% health workers, 19.2% reported that lack of manpower and shortage of stationary were some of the difficulties experienced during implementation of MMSS. Another 7.7% of health workers stated that the MMSS was a fault finding exercise meant to crucify health workers rather than improve service. Only 3.8% of health workers reported that the definition of a maternal death was difficult to apply. The average time taken to complete maternal death notification forms was 20 minutes.

**Table 1:** Knowledge of the maternal mortality surveillance system among health workers in Mutare district, Zimbabwe, 2015

Variable	Frequency n=64 (%)
Correct definition of maternal death	32(50.0)
MMSS objectives	
To provide information that guides actions to eliminate preventable maternal deaths	47(73.4)
To count every maternal death, permitting an assessment of the true magnitude of maternal mortality	11(17.2)
Correct timeframe to report maternal death to the next level	46(71.9)
Type of forms used for MMSS	
Maternal death notification forms (MDNF)	37(57.8)
T5 Forms	1(1.6)
Correct number of notification forms completed for each maternal death	24(37.5)
Correct time frame for conducting a maternal death audit	23(35.9)
How the PMD’s office is informed of a maternal death	
24 hours by phone	9(14.1)
Duplicate copies of MDNF	3(4.7)
Inform the district immediately to inform PMD office	27(42.2)

**Table 2:** Acceptability of maternal mortality surveillance system in Mutare district, Zimbabwe, 2015

Variable	Frequency (n)	Percentage (%)
Participate in MMSS	64/64	100
**Role in MMSS**		
Reporting	50/64	78.1
Investigating	19/64	29.7
Educating community to report maternal deaths	6/64	9.4
Consolidating data	3/64	4.7
**How one learnt to implement MMSS**		
On job training	56/64	87.5
Workshop	6/64	9.4
Formal training	2/64	3.1
Willingness to continue participating in MMSS	64/64	100
Fail to compile MMSS data at times	14/64	21.9
**Reasons for failure to compile MMSS data**		
Unavailability of information	12/14	85.7
Too much workload	4/14	28.6
No stationary	1/14	7.1
Fear of blame	1/14	7.1

**Table 3:** Usefulness of the maternal mortality surveillance system in Mutare district, Zimbabwe, 2015

Variable	Frequency(n)	Percentage (%)
Use MMSS data	51/64	79.7
**If yes, what do you use it for**		
Making informed decisions on maternal health	18/51	35.3
Monitoring performance of public health interventions	13/51	25.5
Capturing maternal mortality statistics	12/51	23.5
Monitoring trends	10/51	19.6
Identifying causes of death	5/51	9.8
**Do you hold meetings to review each maternal death**		
Yes	53/64	82.8
No	1/64	1.6
Never had a maternal death	10/64	15.6
Analyse Maternal Mortality Surveillance data	25/64	39.1
**Have implemented public health action or resolution using information generated from the MMSS**		
Yes	40/64	62.5
No	14/64	21.9
Never had a maternal death	10/64	15.6
If yes, any evidence available	32/40	80

Of all the maternal death notification forms reviewed, 93.8% were completed within seven days of the maternal death and 71.9% deaths had an audit done within seven days of the maternal death. However, it was difficult to determine when the death notification forms reached the provincial office because that information was not recorded anywhere. It was however noted that 96.9% of the notification forms did not reach the national office within the stipulated 30 days of the maternal death because they were signed by the Provincial Maternal and Child Health Officer way after those timeframes. Health workers gave various reasons that could make people fail to report maternal mortality surveillance data timeously to the next level. These reasons were failure to compile report on time because of not understanding some sections of the notification forms (28.1%), breakdown in communication system (23.5%), unavailability of some information/information gaps and community deaths reported late (20.3%), fear of blame (12.5%), health workers not sure of deadlines and lack of transport to investigate deaths in hard to reach areas (9.4%) and unavailability of stationary some of the time (4.7%). Only 53.1% of maternal death notification forms had all the necessary accompanying documents namely: patient's notes, incidence report and maternal death assessment forms. Some maternal death notification forms were incomplete on: education level and parity (6.3%), labour outcome (9.4%); gravida and HIV test result (12.5%). The section which was mostly incomplete was plurality of gestation (68.8% forms), followed by gestational age at booking (37.5%) and thirdly number of antenatal visits which were not completed for 31.3% of maternal death notification forms. Two different types of the maternal death notification forms were found in the field. A total of 19 health facilities were assessed for availability of resources used in the MMSS. Seventeen health facilities had a functional phone on the day of the evaluation. Maternal death case definition was displayed at one health facility only and maternal death notification forms were seen in 14 facilities. Of those facilities with notification forms, 12 had new forms whilst two health facilities had old forms. Maternal death audit forms were seen in five health facilities and 18 facilities had T5 forms. The total average cost of reporting each maternal death in Mutare District was estimated to be $28.65.

## Discussion

The average completeness of maternal death notification forms (MDNF) was 76%. This finding is consistent with findings in a study in Matebelaland North, Zimbabwe were 79% notifications were completed [9]. In a study by Dhege et al in Mashonaland East Province in Zimbabwe, maternal death notification forms were incomplete on education and underlying cause of death [10]. Similarly in this study, 6% and 22% MDNF were incomplete on the same fields respectively. This incompleteness of data can be attributed to the fact that most deaths in Mutare district occur at the provincial hospital and most of the cases are referrals from district hospitals across the province. If the information on demographics is missing when the patient is referred, it might be difficult for personnel at the provincial center to get it from the patient as they come in critical conditions. Incomplete data from surveillance activities becomes difficult to interpret, impossible to validate and ultimately of no use in decision making. The incompleteness of data found in this study is consistent with that found in other studies in Zimbabwe. In a study to evaluate completeness and usefulness of the maternal deaths notification system in Zimbabwe in 2006, the system was found to be incomplete and not standardized [11]. We found two different types of notification forms in the field. This is because new forms were introduced in 2013 and some facilities had not yet received them because priority was given to those facilities that frequently report maternal deaths. The MMSS was found to be useful. This is consistent with a number of MMSS evaluations done that have proved it to be a valuable system. In Ohio's MMSS evaluation, usefulness was a strong component of the surveillance system [12]. We noted that 53(82.8%) health workers reported holding meetings to review each maternal death. It was noted that maternal death reviews help identify challenges and appropriate solutions at the facility level. Effective facility reviews are an important peer-learning process that should remain central to quality of care improvement strategies.

The MMSS was acceptable. All health workers participated in the MMSS and all were willing to continue participating. Staff was committed to the process of maternal death reviews, with routine documentation and reporting. This was evidenced by consistent reporting from all facilities, including zero-reporting from those facilities without any maternal deaths. This is assurance that surveillance is ongoing and health workers are in agreement that no woman should die while giving life. Timeliness of reporting was poor. Failure to compile report on time was the reason cited by most health workers for failing to report data timeously to the next level. Health workers failed to complete maternal death reports on time for various reasons namely; unavailability of information particularly for deaths that occur in the community, no time to compile report due to too much workload and health worker incompetence because some did not know how to complete some fields of the MDNF. It might be difficult to complete the MDNF for the first time especially at primary level facilities where there might be no one to consult. As a result the process will take longer than expected leading to late reporting. Late reporting can lead to delays in appropriate interventions and more women will continue to die from preventable causes. Poor timeliness can also be attributed to poor knowledge of reporting timeframes noted in this study. Other reasons for not reporting maternal deaths data on time were unavailability of transport, too much workload and shortage of staff. To reduce transport costs and when there is no enough manpower to delegate duties to, health workers wait to combine programs before travelling and in the process miss some important deadlines. The increased work burden can be attributed to the staff establishment of health facilities that has remained unchanged for over three decades in an environment with a growing population.

Some health workers report wrong figures to the next level at times. Mostly, health workers report maternal deaths that occur at health facilities. Deaths in the communities are difficult to verify because there is too much movement. As a result if a woman dies outside her area of residence, she may not be reported or may be reported twice leading to under reporting and double reporting respectively. Reporting of wrong figures can also be attributed to the overwhelming workload for health workers and there might be no enough time to do data verification before sending it to the next level. Hence the system would capture wrong figures and the true magnitude of maternal mortality could eventually be distorted. However, the majority of health workers reported that they have never submitted wrong figures. This can be attributed to verification of data done at the health facility level before sending data to the next level. Inadequacy of MMSS data collection instruments was noted. This finding is similar to findings in a study by Valongueri et al (2003) in Brazil [13]. Only 73.7% of health facilities in our study had MDNF and the remaining facilities including Mutare Provincial Hospital did not have MDNF on the day of the evaluation. This can lead to late notification of deaths and ultimately under-reporting. Shortage of stationary can lead to improvising and incomplete data being reported. Unavailability of MDNF in some primary level facilities can be attributed to the fact that some facilities have never reported maternal deaths before; hence distribution of forms to these facilities has been overlooked. Knowledge of what a maternal death is was poor among health workers. This finding can have a negative impact on the reporting of maternal deaths. It means some deaths can go unreported if health workers fail to recognize them because of poor knowledge of the definition of a true maternal death. Poor knowledge of forms used in MMSS seen in this study can be attributed to the fact that some health workers had never encountered a maternal death and therefore had never completed these forms before. We also noted that only a third of health workers knew the number of copies of maternal deaths notification forms completed for each death. The completion of the MDNF is usually done by senior staff and managers at health facilities, therefore junior staff are not usually familiar with these processes. This was confirmed in the study when health workers were asked about the timeframes for MDNF to reach the Provincial office and the majority responded that their superiors manage the reporting process to the higher levels. This can lead to late reporting, if a death occurs in the absence of the senior staff members it might not be reported until they are around to do the reporting.

## Conclusion

Acceptability and usefulness are the strongest components of the maternal mortality surveillance system in Mutare district, whilst timeliness is the weakest component. Completeness of MDNF was not optimal. The system is simple to implement, however, religious objectors are the main obstacle affecting simplicity of the MMSS in Mutare district. Resources for implementing the MMSS were not adequately available in all health facilities. Reasons for not reporting data timeously are failure to compile report on time due to competing programs, unavailability of stationary, fear to report maternal deaths by some health workers and inadequate knowledge of timeframes for reporting. Reasons for reporting incomplete data were unavailability of information especially for community deaths and not understanding some sections of the MDNF. We therefore recommend training of health workers in MMSS, emphasizing on reporting timeframes and definition of a maternal death. MDNF need to be distributed to all health facilities to avoid late reporting and improvising which leads to reporting of incomplete data.

### What is known about this topic

- The highest burden of maternal deaths is in developing countries with sub saharan Africa contributing the majority;
- Maternal deaths are preventable and their occurrence indicates weaknesses in the health-care systems;
- Maternal death notification if mandatory in Zimbabwe.

### What this study adds

- Even though maternal deaths are notifiable in Zimbabwe, a knowledge gap regarding definition and reporting exists among health-care workers;
- Religion remains a significant barrier to effective implementation of the maternal mortality surveillance system;
- There still is a belief among some healthcare workers that the MMSS is a fault finding exercise meant to punish health-care workers.

## Competing interests

The authors declare no competing interest.
